# Fashions and Fancies in Food

**Published:** 1897-08

**Authors:** 


### FASHIONS AND FANCIES IN FOOD.

Thanks to the facilities now afforded to enterprising manufacturers for the advertising of their wares, scarce a month passes without the appearance in the market of a new soap or a new form of bread stuff. Most of the former do indeed possess some distinctive character of merit, whether as regards the enchantment by mechanical or chemical means of their detergent and cleansing power, or the elegance of their prepartion, and the adaptation to the purposes of the toilet; but the popularity acquired by the latter is attributable mainly to their alleged superiority in nutritive value to the old-fashioned wheaten flour, white or brown, which is supposed to be established by analyses signed by chemists of more or less repute, showing that each preparation contains some new fractional percentage of nitrogen above the present in ordinary flours. Endeavors to produce farinaceous, malted, or milk foods suited to the digestion of infants and invalids are deserving of encouragement. But when one considers how large a part of all vegetable and farinaceous foods passes away in the feces without having undergone digestion, and that this portion consists very largely of the denser cortical structures, containing the bulk of nitrogen and phosphates, it does seem no better than trifling to attach so much importance to the retention in or restoration to the flour of a little more of these, whether they consist of the cortex or of the germs, when the meat, eggs and milk—each or all of which enter into the daily dietary of every one—contain the same nutritive principles, albuminoids and phosphates, in incomparably larger proportions, and in far more easily assimilable forms. One of these fancy breads, indeed, shows a remarkable composition in the presence not only of a high percentage of nitrogen, but of such an amount of fat as is found in no cereal or pulse—due, as a microscopic examination clearly shows, to the incorporation of a proportion of linseed meal, which explains, too, the peculiar greenish hue of the bread. Time was when the public judged of the quality of a loaf by the physical characters of color, taste and moisture; but the popularization of science now so general, the "little knowledge" possessed by most "educated" persons, seems to have had the effect of rendering them more susceptible to the representations of pseudoscience, and the blandishments of ingenious manufacturers.—*The Lancet*.

